# Figure recognition and visual attention patterns behind the observation of Palaeolithic art

**DOI:** 10.1007/s41809-025-00170-0

**Published:** 2025-06-08

**Authors:** María Silva-Gago, Marcos García-Diez, Emiliano Bruner, Luis M. Martínez, Felipe Criado-Boado

**Affiliations:** 1 Instituto de Ciencias del Patrimonio (INCIPIT), CSIC, Santiago de Compostela, Spain; 2 Department of Prehistory, Ancient History and Archaeology, Universidad Complutense, Madrid (UCM), Madrid, Spain; 3 Institut Català de Paleoecología Humana I Evolució Social (IPHES), Tarragona, Spain; 4 Museo Nacional de Ciencias Naturales (MNCN), CSIC, Madrid, Spain; 5 Reina Sofia Foundation Alzheimer Centre, CIEN Foundation, ISCIII, Madrid, Spain; 6 Instituto de Neurociencias de Alicante (IN), CSIC-UMH, Alicante, Spain

**Keywords:** Eye-tracking, Perception, Gestalt, Rock art, Vision, Cognitive archaeology, Graphic representation

## Abstract

Art visual processing is a complex cognitive process that involves perception, attention and decision-making. The initial stage of this process is characterized by visual exploration through rapid eye movements which can provide insights into attentional patterns and information processing and can be examined using eye-tracking technology. In this study, we employ eye-tracking to investigate visual perception during the observation of Palaeolithic rock art mostly from the Cantabrian seaboard, aiming to investigate the behavioural mechanisms underlying figure recognition and attentional distribution. This study comprises two experiments—one including entire panels and the other focusing exclusively on single figures—both yielding similar results. Our findings indicate that attention was predominantly directed towards the depicted figures, particularly their heads, rather than other elements within the visual field. Additionally, incomplete figures were perceived as complete, suggesting that figure recognition were influenced by Gestalt principles, internal cognitive models, and other top-down systems.

**Supplementary Information:**

The online version contains supplementary material available at 10.1007/s41809-025-00170-0.

## Introduction

Palaeolithic rock art, as a research category for over 125 years, represents an early manifestation of human creativity and graphic ability, originating at least 65,000 years ago (Lorblanchet & Bahn, 2017; Hoffman et al., 2018). This graphic representation, derived from the replication or reinterpretation of material reality and the development of new representative means of communication. Art, as pictorial communication, constitutes a special category of images; and examining visual art can provide insights into the mechanisms of human vision and perception (Graham & Redies, 2010). Generally, in the visual arts, the creators lead the viewer’s gaze in a given order that they have planned using the composition of the work (Beelders & Bergh, 2020). We could infer this intention to Palaeolithic art, where graphic representations embody a form of human product through which the creator, whether acting individually or as a representative of the social group, transmits ideas or concepts that transcend simple visual materialisation (Pinna & Reeves, 2009).

Viewing art is a complex process that involves perception, attention and decision-making, among other cognitive and affective abilities (Bailey-Ross et al., 2019; Massaro et al., 2012). The formal characteristics of the representation elicit a behavioural response in observers that has been hypothesised to be dissimilar at different periods in prehistoric art (Criado-Boado & Romero, 1993). Palaeolithic art, in particular, constitutes a structured graphic language, designed to be perceived and interpreted by members of the original human group, with the potential for a lasting impact beyond the moment of creation (Hodgson, 2003). This study focuses on this observer’s behavioural response. However, the first process of art perception involves visual scanning through fast eye movements, known as saccades, which are produced between small pauses called fixations (Kowler, 2011). The fixation-saccade system provides insights into patterns of attention and information processing and can be analysed by eye tracking technology (Carrasco, 2011). Fixation patterns reflect overt attention, which is related to directing the eyes actively towards a specific location in space, although information can also be gathered through peripheral vision via covert attention (Rosenholtz, 2016; Wolfe & Whitney, 2015). For the purposes of this study, the term *attention* will refer exclusively to overt attention, understood as the selective focus on an object or spatial area achieved by directing the gaze toward it (Posner, 1980).

Attention, defined as the ability to sustain a cognitive process to resolve a demand despite other distractors (Rueda et al., 2023; Shiferaw et al., 2019), can be influenced by bottom-up factors, such as colour, brightness, texture, and other salient features, or by goal-oriented and executive functions known as top-down factors (Baluch et al., 2011; Chica et al., 2013; Connor & Egeth, 2004; Katsuki & Constantinidis, 2014). Specifically, in art perception, these mechanisms interact together to direct attention towards the most salient features while also aligning with the creators intended focal points (Beelders & Bergh, 2020). Therefore, attention works as a selective filter, guiding visual focus to relevant information. Attention likely underwent a remarkable enhancement and specialization in the human genus, and as far as we can infer by changes in technological complexity, social organization, and fronto-parietal neuroanatomy (Bruner & Colom, 2022). In this sense, focused and executive attentional mechanisms, namely, the ability to control attention to ongoing cognitive processes, avoiding distractions and targeting to achieve a specific goal (Engle et al., 1999; Geva et al., 2013), contributed to evolve an intentional, sustained, and aware attentional process (Bruner, 2024). This attentional network largely relies on visual processing, which is the primary source of information in primates (Atkinson, 2008; Matsuno & Fujita, 2009).

The selection and processing of relevant information is a prediction system that involves the management of external salience factors (bottom-up) and internal sources of information (top-down). According to active inference models of cognition, the brain continuously generates predictions to minimise errors between perceived information and previous experiences, enabled by actions such as eye movements (Clark, 2023; Constant et al., 2021; Friston et al., 2009). In addition to the observation of salient locations, saccades optimise selective visual sampling through continuous adjustments, with fixations directed toward the most significant areas of a scene (Shiferaw et al., 2019). This process as a whole allows for a comprehensive understanding of what is being observed.

Palaeolithic art probably encodes messages and discourses that influence individual responses. Interpretation of these messages is conditioned by prior experience in visual comprehension, through a proper understanding of the relationship between symbol and meaning, and the perception/recognition of the symbols themselves (Pinna & Reeves, 2009). The composition, defined as the combination of elements within a painting, significantly influences visual behaviour, even if its meaning is not fully understood (Beelders & Bergh, 2020; Sancarlo et al., 2020).

As mentioned, visual behaviour can be analysed by eye-tracking technique. The application of this method has a quite longstanding tradition within the field of art studies (e.g., Bailey-Ross et al., 2019; Buswell, 1935; Massaro et al., 2012; Molnar & Day, 1981; Rosenberg & Klein, 2015; Savazzi et al., 2014; Serino & Villani, 2015; Yarbus, 1967) and have also been applied in archaeological research (Criado-Boado et al., 2019, 2023; Silva-Gago & Bruner, 2023; Silva-Gago et al., 2021, 2022), indicating that visual attention patterns differ according to both biological and cultural changes. Regarding Palaeolithic art, some studies have analysed the visibility and positioning of depictions from multiple approaches (Garate et al., 2023; Intxaurbe et al., 2022; Ochoa & García-Diez, 2018; Rivero et al., 2024; Wisher et al., 2023b, c) while others focused on exploring the role of fire illumination in its visualization (Medina-Alcaide et al., 2021; Needham et al., 2022). The spatial distribution of paintings across different artworks seems to be determined by the cave’s morphology, which naturally provides forms and shapes for who created the depictions (Wisher et al., 2023b), and in some cases, they are located in areas highly visible to a large audience (Garate et al., 2023; Intxaurbe et al., 2022; Ochoa, 2017). On the other hand, a computer-based program developed for facial expressions showed that the anatomical parts most frequently depicted in rock engravings are the same as those considered most salient in photographs of present-day animals (Meyering et al., 2020). However, none of these studies have quantitatively analysed the visual attention directed to cave paintings.

Despite all the approaches developed in specific case studies using other methodologies, the distribution of attention within a panel or depiction has not been quantified in a broader number of caves. The present study is conducted within the framework of the XSCAPE Project on Material Minds (ERC—Synergy Grant). This interdisciplinary project investigates the relationship between the material world and human cognition, with a particular focus on eye-tracking methodologies applied to the analysis of material culture. In this case, we applied this technology in a wide selection of the most iconic caves with Palaeolithic art. The first study was exploratory and involved a wide range of figurative and abstract representations across entire panels, while the second experiment includes only single figures and aimed to assess whether and to what extent incomplete figures are recognised and observed as if they were fully represented. Our research work focused on the relationship and perceptual hierarchy existing between depictions made by past humans (graphic motifs) and natural forms of the cave (wall elements of geological origin such as fissures, reliefs, etc.); and secondly, on the analysis of the perceptual hierarchy of the different anatomical regions that articulate a zoomorphic form. We test the null hypotheses that (i) attention depends on saliency of the cave wall visual features, and (ii) the anatomic representation of the figure does not influence the patterns of visual exploration.

## Experiment 1

### Material and methods

*Participants*. An opportunistic sample of 12 participants (5 females and 7 males) recruited from social media took part in the study. All subjects had normal or corrected-to-normal vision and they were aged between 19 and 55 years old (42 ± 11 years). Most of them lived in an urban environment (92%) and had a university education (83%). All gave informed consent for their participation in the study, which was approved by the ethics committee of the Spanish National Research Council (CSIC).

*Stimuli*. Twenty-six photographs of depictions from 15 iconic Palaeolithic caves on the Cantabrian seaboard and France (El Castillo, Covalanas, Llonín, Trescalabres, La Pileta, Altamira, Las Chimeneas, Covaciella, Ekain, La Garma, Las Monedas, La Pasiega, Sotarriza, Tito Bustillo and Chauvet) were used in the analysis (Fig. 1 of supplementary material). Five photographs of real animals (bisons and horses) were used as control images. Some depictions were single-figure as opposed to multiple panels (3 with two motifs, 1 with three motifs and 1 with seven motifs). Likewise, animal motifs were the most frequent theme (a total of 18 including bisons, deers, horses, aurochs and goats), five pictures with signs (rectangular, quadrangular, sinuous, grille, couplet, radiate, grill and lines), and two anthropomorphic or indeterminable shape. The degree of anatomical completeness in the animal figures has also been considered (13 complete and 5 incomplete) and their chronology (when it has been possible to determine it with certain guarantees: 7 pre-Magdalenian -approx. between 40.000 and 18.000 BP- and 9 Magdalenian -approx. between 18.000 and 12.000 BP-). The distinction between these two categories is well-established in the literature and reflects two chronological phases. In general, Pre-Magdalenian figures are primarily defined by outlined forms, minimal anatomical detail, occasional disproportions, and a simplified stylistic approach. In contrast, Magdalenian representations exhibit greater proportional accuracy and a more naturalistic style.

**Fig. 1:**
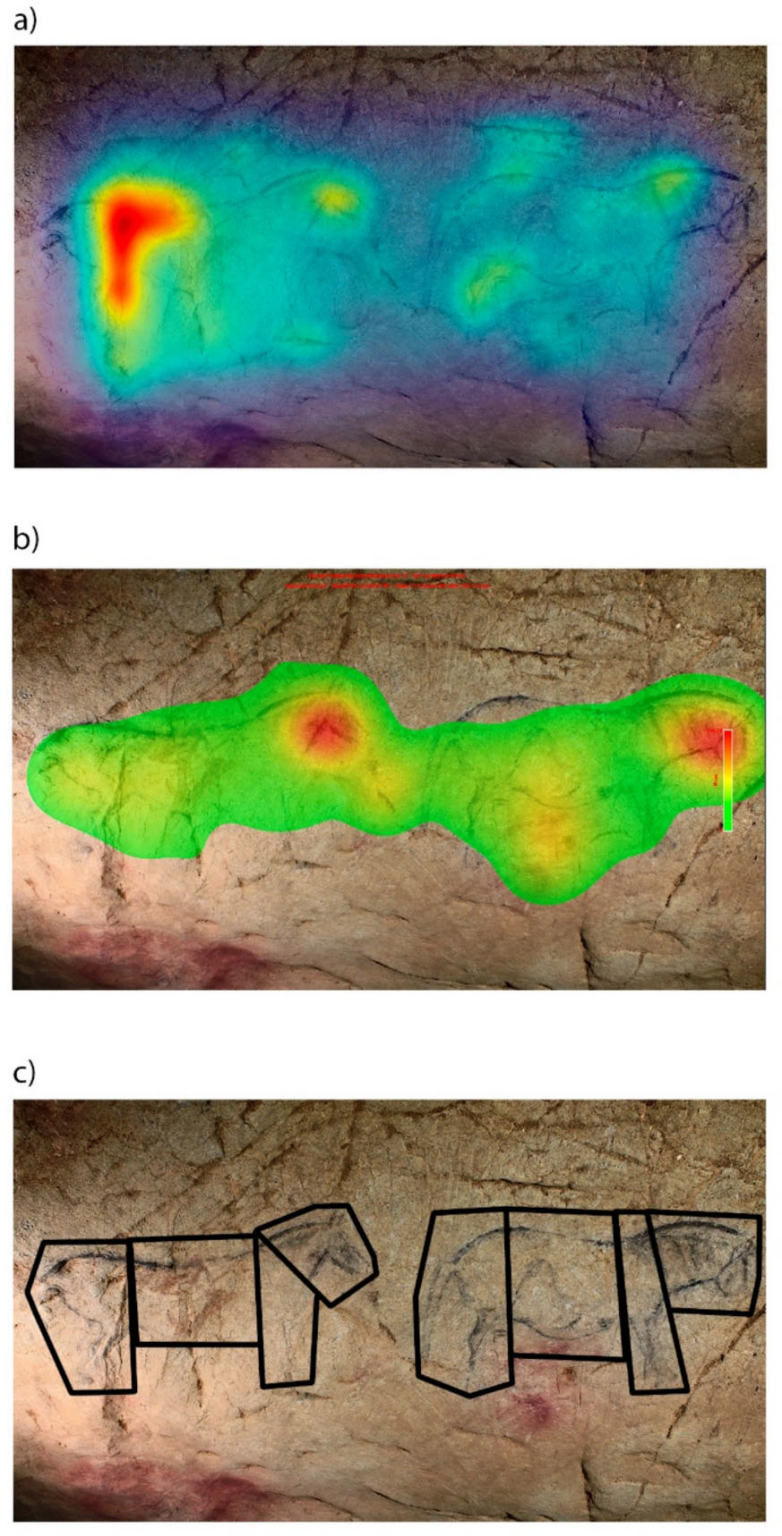
An example of saliency computation map (**a**) and fixation map (**b**). While saliency map highlight in red some fissures of the cave and angled areas, fixation map indicate that the most observed points are the horses’ heads (also in red). Below, an example of AOIs design (**c**) of the same panel according to the anatomical regions represented (right) and estimated when missing (e.g. anterior region of left depiction)

*Procedure*. Participants were asked to freely visually explore a set of 26 images for 10 s each while resting their heads on a chin rest in front of a 28″ monitor. The distance between the participant and the screen was 115 cm. Eye movements were recorded using the eye tracker device Eye Link 1000 Plus (SR Research, Canada), from the Material Minds Lab (MML), a facility provided by the XSCAPE project in the Institute of Heritage Sciences (INCIPIT—CSIC). Pupil position was sampled at 500 Hz. Eye Link 1000 Plus is a desk-mounted eye tracker consisting of a camera that automatically focuses on the subject’s eyes, illuminated by an infrared system. Stimulus presentation was built by a Python code which randomize the images for each participant. All participants viewed the same set of pictures, which were standardized to same size and resolution (1920 × 1080, 300 dpi). Eye position was calibrated by the fixation of nine predefined dots, sequentially presented on the monitor. Each trial started with a drift correction to ensure that calibration was valid. The calibration was repeated during the experimental session whether the participant left the chin rest for any reason or whether the calibration was not correct according to drift point validation.

*Data analysis*. First, the areas of greatest visual saliency and therefore likely to attract attention were calculated by graph-based visual saliency (GBVS) computation algorithm using Matlab software (Fig. 1a; also see Silva-Gago et al., 2022). The algorithm decomposed the original image into distinct visual feature channels, such as colour, luminance, and orientation, and then computed contrast values at each location within the image for each of these individual feature channels. Then, different areas of interest (AOIs) were defined based on these most identified salient locations (called *saliency AOI*), other elements in the cave such as fissures, calcite formations and other changes in the morphology of the support (called *cave AOI*), as well as the *depiction*. Hereafter, saliency features refers to the most salient areas computed (in red colour) by the GBVS algorithm. Furthermore, some AOIs were defined based on the main anatomical regions of the figures, namely *head, anterior*, *body* and *posterior* (Fig. 1c). Missing anatomical regions of incomplete depictions were not considered and were not included in any AOI. We computed the dwell time (in milliseconds) relative to the size of each AOI (in pixels) as a measure of fixation density over an area (Silva-Gago et al., 2021). This variable (relative dwell time, DT_REL) standardizes the amount of attention directed to each AOI regardless of their size. According to this methodological approach, fixation count correlates with dwell time (Silva-Gago et al., 2022), thus it has been decided to show only the results of the second. Fixation maps were generated by Eye Link Data Viewer to visualize the most observed areas (Fig. 1b). Mann–Whitney test, Dunn test and Kruskal–Wallis test were run between AOIs and depictions in order to test differences. Bonferroni correction was applied in all tests. Percentages of fixation time were computed according to the fixation time directed the AOIs defined in the stimuli, excluding saccade movements. All analysis were conducted using R 4.4.1 (R Core Team, 2024), the ggplot2 (Wickham, 2016) and ggstastsplot packages (Patil, 2021).

### Results and discussion

**Fig. 2:**
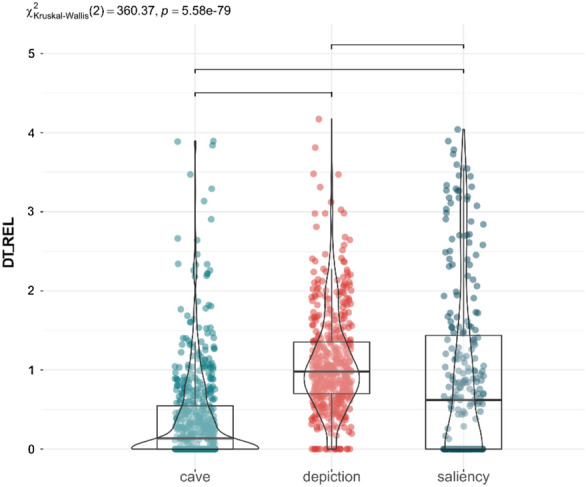
Distribution of Relative Dwell Time for each AOI: cave fissures, calcite formation and other morphological changes (blue), depiction (red), computed saliency areas (grey). Upper bars show significant differences (p < 0.05, Bonferroni correction)

**Fig. 3:**
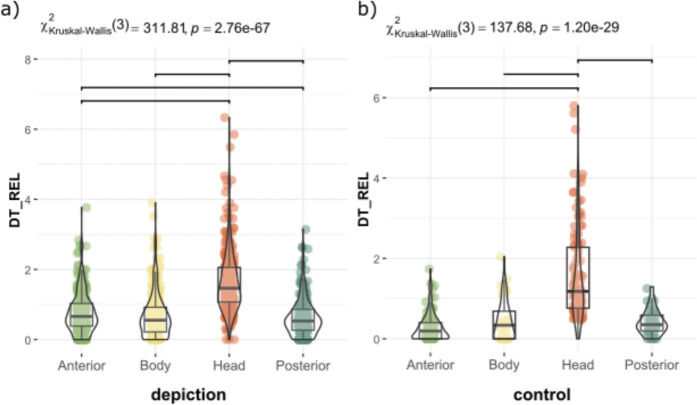
Distribution of Relative Dwell Time for each AOI in depictions (**a**) and control images of real animals (**b**). Upper bars show significant differences (p < 0.05, Bonferroni correction)

Considering all the pictures, Fig. 2 shows the distribution of DT_REL across each AOI, demonstrating significant variability. Many visually salient areas received no fixations, while the depiction attracted more attention than both the salient regions of the photograph and other cave features, such as fissures (p < 0.001). The painting accounted for 86% of the total fixation time, in contrast to only 8% for prominent areas and 6% for cave features likely to attract visual attention. The algorithm identifies the brightest or angular areas as the most salient ones, sometimes also coinciding with cracks or ridges where several fractures converge. However, they have barely been observed in comparison to the depiction even when they were used as an element of the figure (Wisher et al., 2023b). This is consistent with previous studies showing that more sensory salient features, namely the areas considered salient by computation algorithms, receive less attention when there are other underlying purposes that are influencing attention (Criado-Boado et al., 2019; Silva-Gago et al., 2021) (see Fig. 3).

In zoomorphic paintings, the head was the most frequently fixated anatomical region (p < 0.001; Fig. 2a). Human attention is inherently drawn to faces and heads (e.g. Cerf et al., 2009; Gregory et al., 2015; Hietanen, 1999; Leppänen, 2016; Langton et al., 2000), due to their social and communicative significance. In the current study, this distribution of visual attraction was similar to the control images of real animals (Fig. 2b), with no statistically significant differences between the observation of real horses or bisons and the cave representations (p > 0.05). The similarity of the observation of the animals and the paintings could be related to the fact that the figures on the cave walls actually depict the animals in the environment. They are representative images of materiality.

Regarding the sequence of visual attention, the head accounted for 71% of the total fixation time, while the other anatomical regions accounted for 29%. Initial fixations were most often directed at the centre of the stimuli, typically corresponding with the body of the Fig. (58% of cases), followed by the head, which was the next most frequently fixated region (85% of cases). The head is one of the most discriminant or recognisable parts of an animal (Meyering et al., 2020) so most attention is directed there to facilitate understanding of what is being observed.

**Fig. 4:**
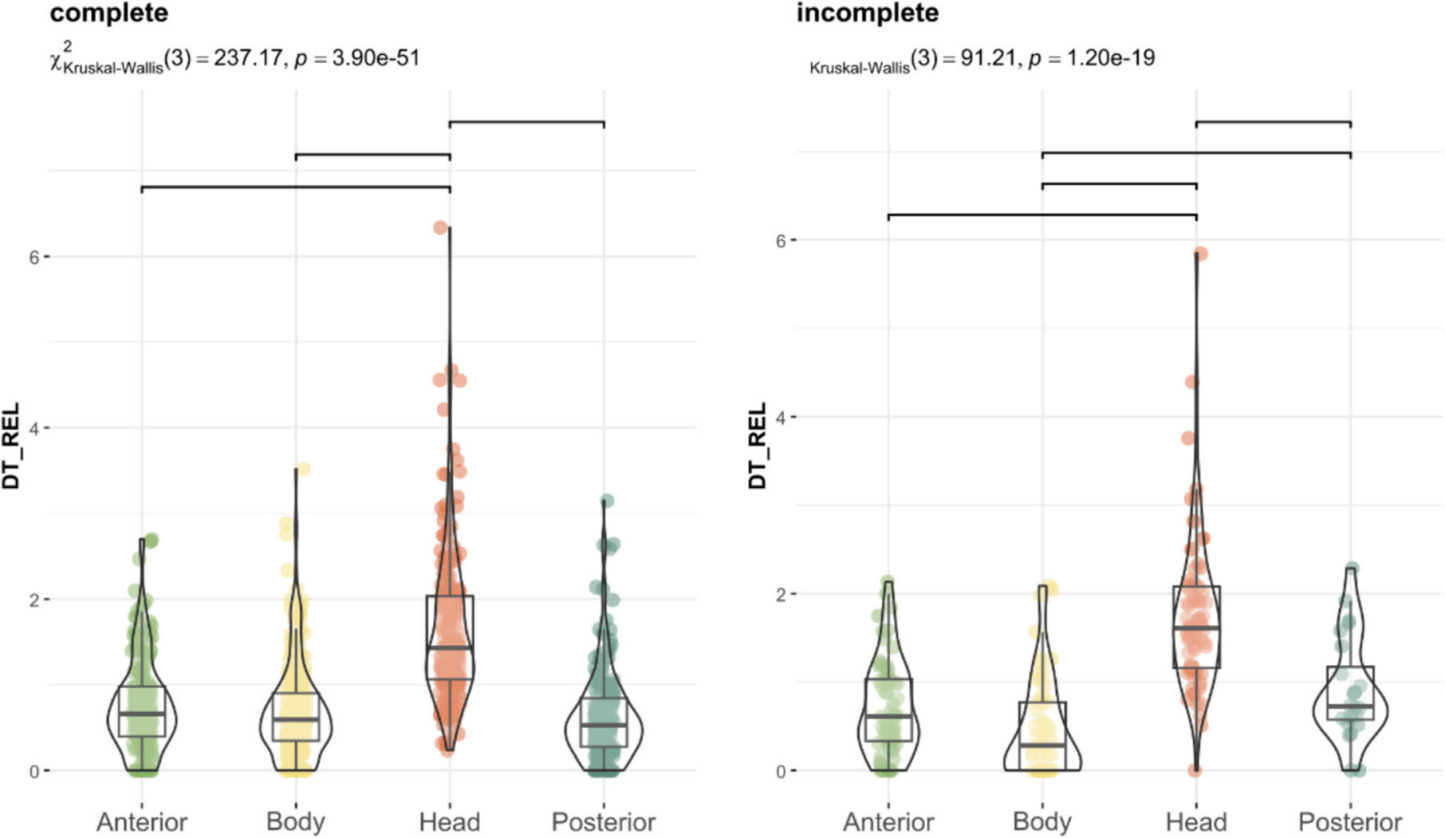
Distribution of Relative Dwell Time for each AOI in complete (left) and incomplete (right) depictions with figurative motifs. Upper bars show significant differences (p < 0.05, Bonferroni correction)

When comparing incomplete and complete figures, the distribution of visual attention was similar, the head remained the most observed region, displaying significant differences from the other anatomical regions (p < 0.001). Nonetheless, complete figures attracted more fixations on the body and incomplete depictions on hind legs (Fig. 4).

**Fig. 5:**
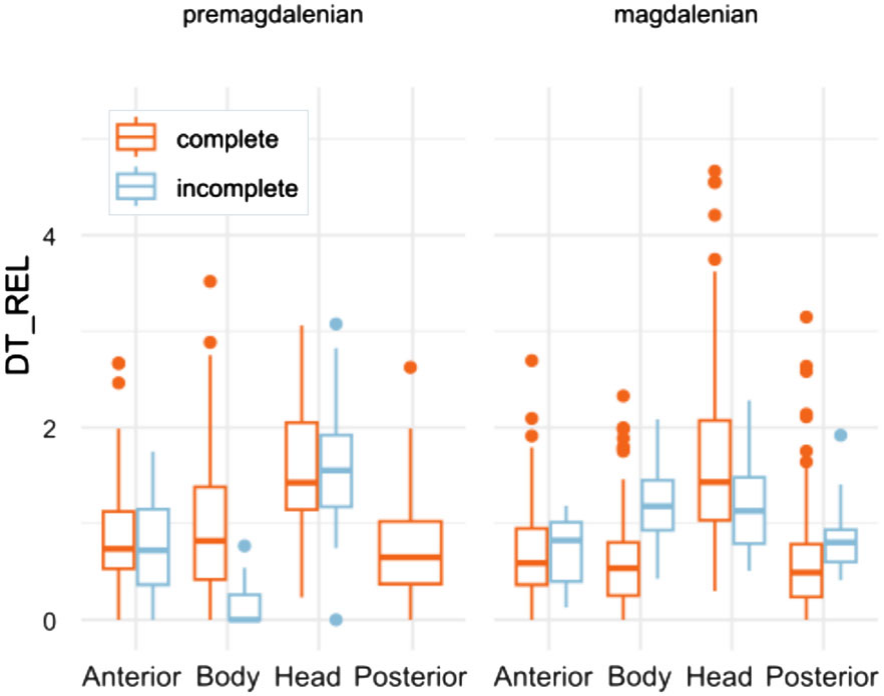
Distribution of Relative Dwell Time for each AOI in Premagdalenian and Magdalenian depictions. Panels with no clear chronological association were excluded from analysis

Visual behaviour was also similar in different periods (Fig. 5). There were no differences between Magdalenian depictions and paintings from previous chronologies (p > 0.05). The head was the main focus of attention, regardless of the degree of completeness and the chronological period. It is also noteworthy that the attention in the incomplete Magdalenian depictions was more equally distributed among the different anatomical regions, particularly the head and the body. In summary, the focus on the head region is preferential to other anatomical regions, whether the figures are complete or incomplete, and throughout the Palaeolithic art's temporal sequence.

## Experiment 2

### Material and methods

*Participants*. An opportunistic sample of 29 participants recruited from social media took part in the study, including 19 females and 10 males (40 ± 13.3 years old). Most of them lived in an urban environment (86%) and all had university studies. All subjects had a normal or corrected-to-normal vision and gave informed consent for their participation in the study, which was approved by the ethics committee of the Spanish National Research Council (CSIC).

*Stimuli*. A different sample of twenty-six photographs of depictions from 20 iconic Palaeolithic caves on the Cantabrian seaboard and France (El Castillo, Covalanas, Llonín, Trescalabres, La Pileta, Altamira, Las Chimeneas, Covaciella, Ekain, La Garma, Las Monedas, La Pasiega, Sotarriza, Tito Bustillo and Chauvet, Lascaux, Cougnac, Le Portel and Niaux) were used in the analysis (Fig. 2 of supplementary material). Three drawings of Postpaleolithic art and three pictures of prehistoric vessels were used as distractor images, and were excluded from analysis.

All depictions were single-figure and showing animal motifs (bison, doe, deer, horse, auroch, goat and bear). The sample of animal figures included 13 complete and 13 incomplete depictions. Most of the them were faced to the right (18), black colour was predominant in 11 panels, and red in 9 depictions (6 pictures include more than one colour). When it was possible to determine the chronological adscription, 10 panels were pre-Magdalenian (approx. between 40.000 and 18.000 BP) and 13 paintings were Magdalenian (approx. between 18.000 and 12.000 BP).

*Procedure*. Participants were asked to observe 32 pictures for 10 s each following the same experimental set up as in Experiment 1 above. Eye movements were recorded using Eye Link 1000 Plus (SR Research, Canada) sampling pupil position at 500 Hz. Stimuli were presented using a python code which randomize the images for each participant. All participants viewed the same set of pictures. Eye position was calibrated by the fixation of nine predefined dots and each trial started with a drift correction to ensure that calibration is valid.

**Fig. 6:**
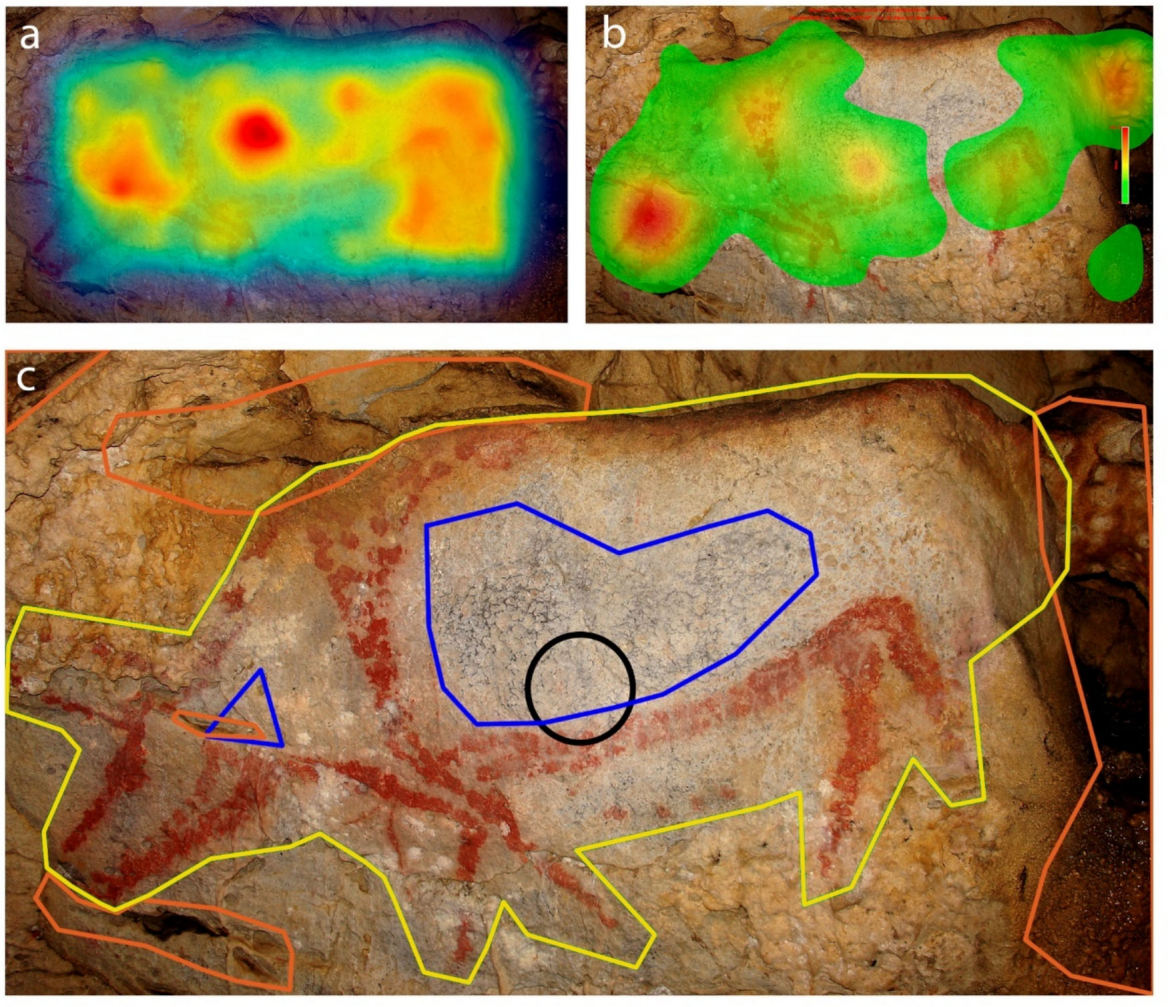
Example of saliency map (**a**), average fixation map (**b**) and AOI design: saliency AOI (blue), cave AOI (orange), depiction (yellow) and centre of the image (black). Saliency AOI was described according to the most salient areas calculated in the saliency map (the reddest locations). When one fixation belongs to more than one AOI, it was included in both AOIs due to the impossibility to discriminate

*Data analysis*. Analysis was similar to previous Experiment 1. Saliency maps were generated using GBVS algorithm and Matlab software. The same AOIs were described based on computed saliency regions, cave characteristics and the depiction, as well as the anatomical regions of the figure (head, anterior, body and posterior). A depiction is considered incomplete when one of these anatomical regions is missing. The main difference compared to Experiment 1 lies in the estimation of the missing region in the incomplete depictions according to the size of the depicted animal. While in Experiment 1, the missing regions of incomplete depictions were not considered, in Experiment 2, that missing regions (for example, the posterior legs) were estimated in order to know if the attention was directed to that area even if it was not represented (Fig. 1c). The centre of the images was also considered as an AOI (Fig. 6). We calculated the fixation time relative to the size (DT_REL) as the main variable to examine the allocation of attention across the panels (Silva-Gago et al., 2021). Fixation maps were generated by Eye Link Data Viewer to visualize the most observed areas. Mann–Whitney test, Dunn test and Kruskal–Wallis test were run between AOIs and depictions in order to test differences. Bonferroni correction was applied in all tests. All analysis were conducted using R 4.4.1 (R Core Team, 2024), the ggplot2 (Wickham, 2016) and ggstastsplot packages (Patil, 2021).

### Results and discussion

**Fig. 7:**
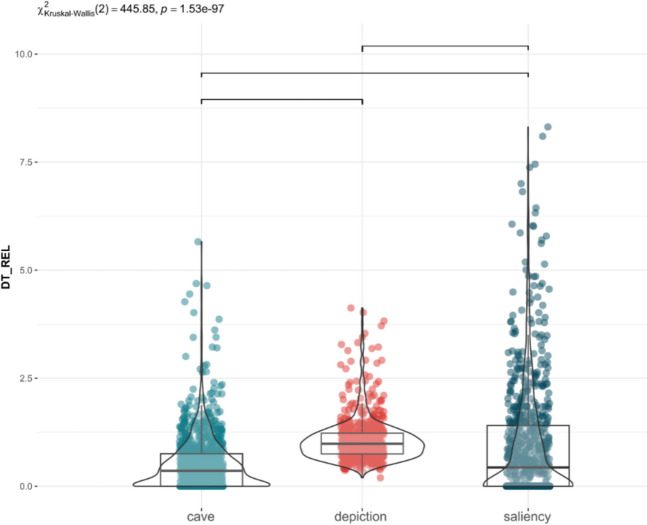
Distribution of Relative Dwell Time for each AOI: cave fissures, calcite formation and other morphological changes (blue), depiction (red), computed saliency areas (grey). Upper bars show significant differences (p < 0.05, Bonferroni correction)

Considering all the figures, the depiction attracted significantly more attention than the visually computed salient areas of the panels and other cave features (p < 0.001; Fig. 7). The distribution of attention across saliency areas exhibited the greatest variability. The painting itself accounted for 90% of the total fixation time, whereas areas considered prominent were observed for only 7%, and cave features likely to attract attention were observed for just 3%. Hence, the painting received most of fixation time regardless of whether it uses the cave's topography or not, in contrast to suggested in previous studies when the painting was removed (Wisher et al., 2023c).

**Fig. 8:**
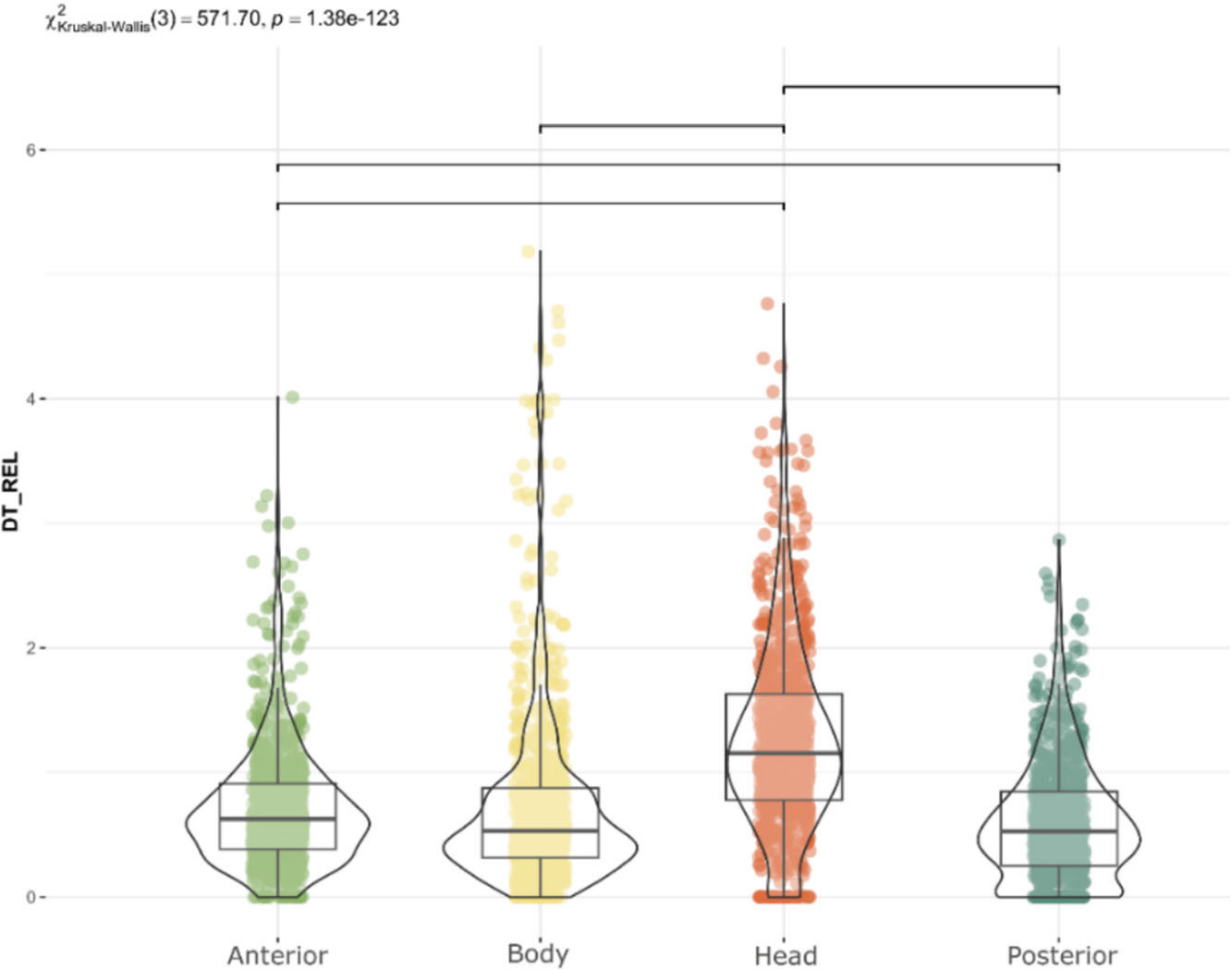
Distribution of Relative Dwell Time for each AOI. Upper bars show significant differences (p < 0.05, Bonferroni correction)

**Fig. 9:**
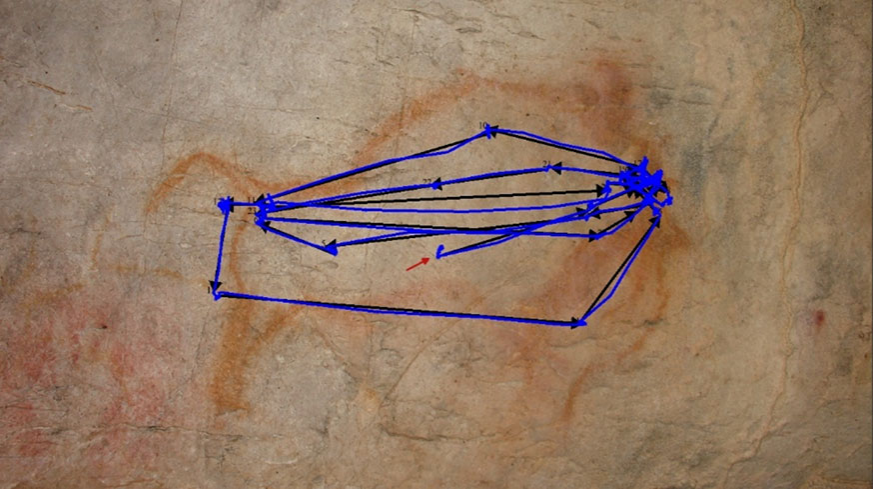
Example of a participant’s scanpath (blue) and saccades direction (black arrows). The initial fixation was directed to the centre of the image (red arrow), while the second and the third was located in the bison’s head (following the black arrows)

**Fig. 10:**
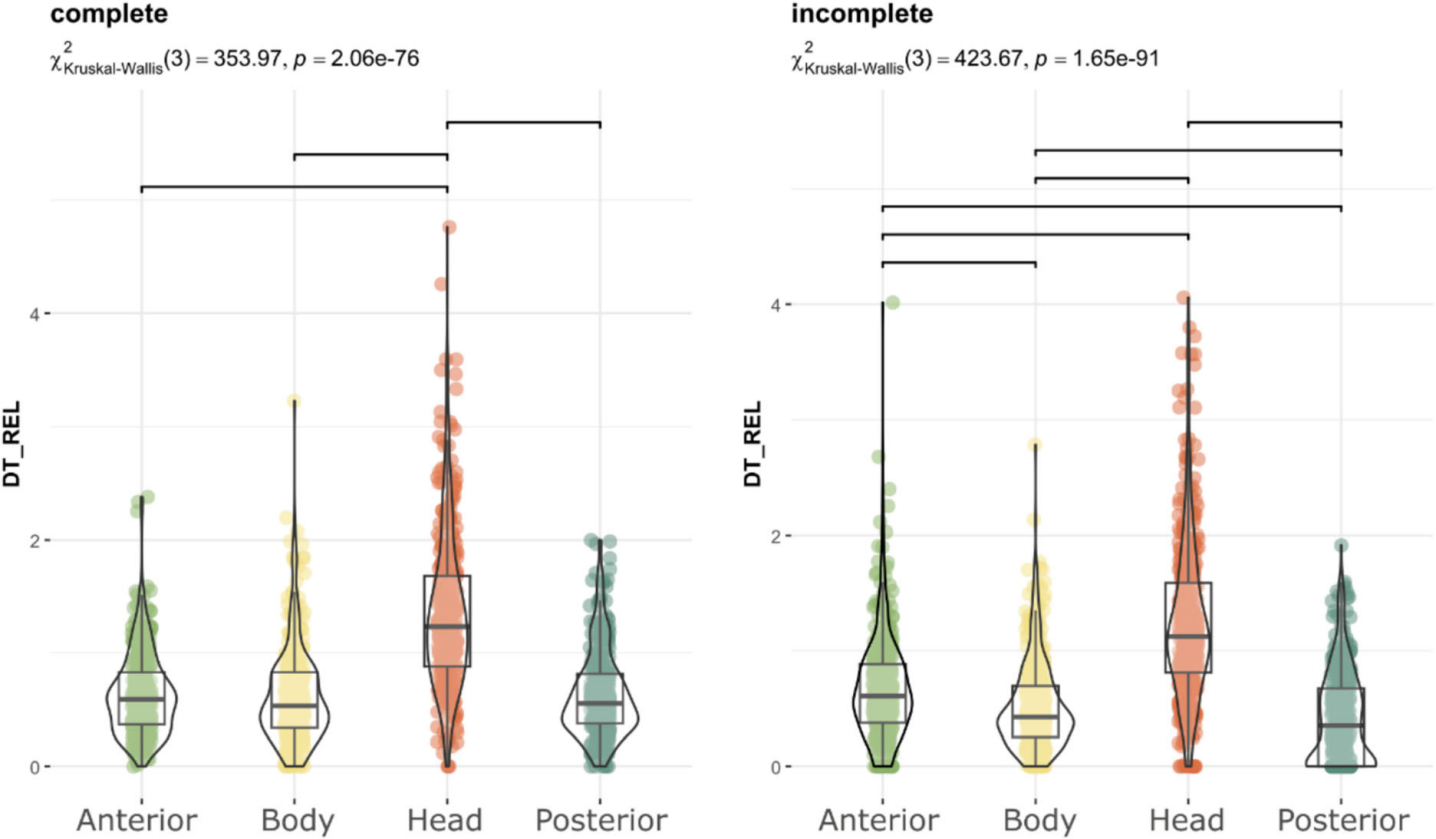
Distribution of Relative Dwell Time for each AOI in complete (left) and incomplete (right) depictions with figurative motifs. Upper bars show significant differences (p < 0.05, Bonferroni correction)

Regarding the anatomical regions, the head received the highest proportion of fixations (p < 0.0001; Fig. 8). After the initial fixation, which was predominantly directed at the centre of the image due to centre bias (Doran et al., 2009; Ioannidou et al., 2016; Le Meur & Liu, 2015; Tatler, 2007), and coinciding with the body of the depicted animal, the head was the next most frequently observed area, attracting 40% of fixations (Fig. 9). The anterior region accounted for 20% of fixation time, the body 18%, and the posterior region 17%, while the centre of the image received only 5%. As previous studies have shown (Cerf et al., 2009; Hietanen, 1999; Langton et al., 2000; Meyering et al., 2020), the head is the region most observed and the most suitable for identifying an animal depicted. When comparing the overall distribution of visual attention of complete and incomplete depictions, the results were similar, suggesting that the degree of completeness did not influence the observation of the painting and the recognition of the animal represented (Fig. 10). Likewise, the sequence of gaze direction was similar, with the head being the following fixated area after the first fixation directed to the centre of the picture.

**Fig. 11:**
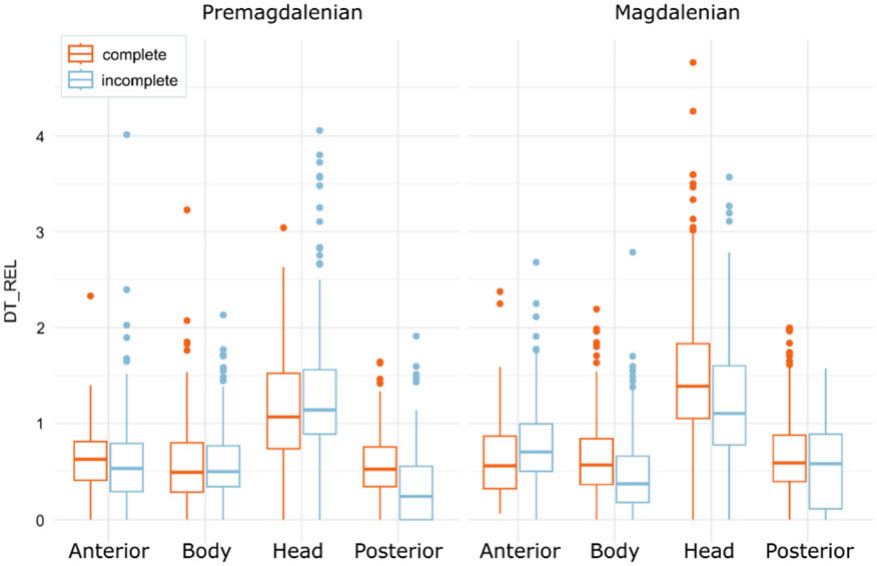
Distribution of Relative Dwell Time for each AOI in Premagdalenian and Magdalenian depictions. Panels with no clear chronological association were excluded from analysis

Furthermore, in a chronological comparison, the fixation pattern was similar, the head is the most observed region. However, significant differences were found in the fixation time on both extremities (p < 0.001), while the body and head showed similar values. When examining by degree of completeness, complete Magdalenian figures were generally more observed, particularly in the head region, compared to pre-Magdalenian paintings. On the other hand, in incomplete figures, the head showed similar fixation times, but there were differences across the rest of the body, namely, incomplete Magdalenian figures received more attention on the posterior regions, whereas pre-Magdalenian figures were more frequently observed on the body. Furthermore, in Magdalenian period, there are more differences between complete and incomplete depictions than in previous chronologies (Fig. 11). This difference may be related to the greater degree of anatomical detail in the Magdalenian paintings (García-Diez et al., 2016; Ochoa et al., 2019; Ruiz-Redondo, 2016), causing attention to be more distributed and focused on those finer details.

Other factors, such as colour (black or red), laterality (whether the animal faced left or right), or drawing technique (whether the figure was filled in or depicted only in outline), did not show any significant differences in fixation patterns (p > 0.05). The fact that these factors did not influence visual perception may be evidence that the main information extracted was related to the semantic meaning of the painting.

## General discussion

Interest in Palaeolithic art has a long-standing tradition within archaeology and the study of human evolution, but only recently new approaches focused on visual perception have been applied (e.g. Garate et al., 2023; Intxaurbe et al., 2022; Meyering et al., 2020; Wisher et al. 2023a; Wisher et al., 2023b, c). However, no study had quantitatively assessed visual attraction across a wide variety of panels from different caves. This study consists of a preliminary investigation of gaze direction during the observation of a representative selection of rock art. We present two experimental studies in which eye tracking was used to record the visual behaviour elicited by several Palaeolithic paintings. Experiment 1 included both figurative and non-figurative representations, while Experiment 2 focused on single zoomorphic figures, including more sample to confirm the hypothesis. Although both experiments were processed and analysed using slightly different methodologies (the areas of interest design differs), the results consistently highlight a main focus on figures, particularly on the heads of animals, instead of other elements of the visual field. Therefore, results are discussed in relation to these two issues.

### Relationship and perceptual hierarchy between depictions and cave’s topographic features

Previous studies have suggested a strong relationship between cave topography and the depicted figures (Fano et al., 2022; Garate et al., 2023; Intxaurbe et al., 2022; Jouteau et al., 2019; Robert, 2017; Sakamoto et al., 2020; Wisher et al., 2023b, c). Specifically, some studies explored the visual perception of cave walls in the absence of the paintings themselves, proposing that the surface’s topography may suggest the shape of the depicted animal (Wisher et al., 2023b). However, the findings of the present study indicate that cave morphology had a limited influence on the attraction of visual attention when the depiction is present. Likewise, this effect is enhanced when panels or walls are considered in an overall view (Fig. 2) instead of single figures (Fig. 6). In cases where the painter did not integrate the cave's natural features into the artwork, the depiction consistently drew attention over fissures and other morphological elements.

On the other hand, some figures in both experiments did incorporate cave features as part of the depiction. While fixation maps revealed no substantial focus on these salient regions of the cave, these areas exhibited higher attention values compared to the median. In other words, the fixation maps did not indicate considerable attention directed toward those areas. However, a slight quantitative trend is noticeable when compared the dwell time directed to the different AOIs in two specific panels: a doe from Altamira and a bison and horse from Chimeneas. This suggests that, although cave topography might contribute to the overall composition, its significance is more pronounced for the painter during the creation process than for the observer during visual engagement. The topographic features appear to be embedded within the whole perception of the painting rather than serving as specific points of attention (Criado-Boado and Penedo-Romero, 1993; Sakamoto et al., 2020). In sum, taking advantage of the cave's morphology seemed to be more significant for the painter than for the observers.

### Perceptual hierarchy of the anatomical regions in zoomorphic depictions

Selective attention is the mechanism by which information from visual inputs is selected and can therefore be directed to a particular area to prioritise the processing of sensory information within the visual field (Chapman and Stormer 2024). Consequently, fixations are directed towards the most informative regions of a visual scene, becoming more dispersed as the perceptual relevance of information increases to facilitate selective extraction and minimize redundancy (Shiferaw et al., 2019).

When analysing attention directed to different anatomical regions of animals, the head had consistently been the most observed area. There is a general tendency to look at the head and face of figures, a behaviour shared by humans and primates (Rolls et al., 2000; Webster and MacLeod, 2011; Yu et al., 2024). In this case, the attention directed to the head could be associated with semantic processing, defined as the ability to associate and access the meaning of stimuli (Cocquyt et al., 2019; Mudrik et al., 2014), animal identification or the geometric complexity of this region compared to the rest of the body (Chapman & Störmer, 2024; Faghel-Soubeyrand et al., 2024). The head is relevant from an informative point of view because allows the observed figure to be recognised and correctly identified, and commonly corresponds to the most represented regions in rock art (Meyering et al., 2020). On the other hand, it is generally the area with the highest number of strokes, curves and lines, which means that it becomes a focus of information at a geometric and shape level. This preference for curvatures is a universal human behaviour also found in great apes (Gómez-Puerto et al., 2016; Munar et al., 2015) and it is associated with an efficient way of extracting the most meaningful elements from visual field (Shevelev et al., 2003; Sigman et al., 2009).

The head constitutes a fundamental point in the perception of paintings, whether due to its geometric organization or its thematic significance, even when not fully depicted. For instance, participants also looked for the head in incomplete figures. A comparison of complete and incomplete depictions revealed a similar distribution of attention, namely, the head was the most observed region, followed by the anterior region. Since these areas are the most diagnostically significant parts of an animal (Meyering et al., 2020), this finding highlights the importance of these regions in facilitating the recognition or enhancing the interpretative meaning of painted figures (Pinna & Reeves, 2009). Therefore, the location and detection of the figures’ head could be essential to guide visual exploration patterns during the observation of the paintings, as it was probably intended by the creators. The importance of identification appears to be reinforced by the fact that factors such as colour or figure orientation did not affect visual behaviour.

Among the many factors conditioning human vision are Gestalt principles, which explain how visual information is perceived and organized (Wertheimer, 1938; Kofka, 1935). These principles play a key role in the recognition of incomplete figures, allowing to “fill in the gaps” and perceive a complete figure despite missing elements. This process is based on a series of laws: closure, continuity, proximity, and similarity (Spillmann, 2006). Through the Gestalt laws, the human visual system combines contours, corners and other simple disconnected visual elements as unified and coherent animal figures (Hu & Niebur, 2017; Massaro et al., 2012; Onians, 2024; Persike & Meinhardt, 2016; Yan et al., 2018; Zupan & Gvozdenović, 2023).

Simultaneously, the anatomical parts represented might contain the information necessary to understand what is being observed due to visual concepts stored in memory, also known as the inner model, observers had of the animals. The internal model is constantly updated through predictions and prediction errors, modelling by eye movements and attentional changes (Clark, 2023; Constant et al., 2024; Friston et al., 2009; Moberget et al., 2014; Shiferaw et al., 2019). In other words, once an observer had encountered a horse, an internal model of its structure is integrated. When observing a depiction, even if it consists only of fragments resembling a horse, the brain generates predictive hypotheses to confirm its identification. These predictions are tested and refined through eye movements and attentional processes, enabling recognition despite incomplete or partial visual input. Although these questions are based on neuroscientific approaches, similar interpretations have been proposed from archaeology and ethnography, suggesting that incomplete paintings were perceived as whole figures through the internal models constructed by prehistoric observers (Clottes and Lewis-Williams, 1998).

Furthermore, the focus on the head region was predominant over other anatomical regions throughout the whole temporal sequence of Palaeolithic art. In general, there were no differences between pre-Magdalenian and Magdalenian depictions. However, some detailed regions such as extremities were more observed in Magdalenian figures, maybe associated with an enhanced representation of these features (García-Diez et al., 2016; Ochoa et al., 2019; Ruiz-Redondo, 2016). We hypothesised that this visual prevalence of anatomical details, in some cases, could be related to the location of the Magdalenian paintings in elevated locations (Itxaurbe et al., 2022) in order to facilitate the understanding and identification of the figure. Nevertheless, further studies focusing on chronological differences will confirm this hypothesis.

## Conclusion

Palaeolithic graphic representation emerges from the replication or reinterpretation of material reality and the development of new representational systems for communication. It exemplifies the inception of art, as graphic language, and the development of abstract concepts, which are beyond the scope of this study. This article focused on the behavioural and biological phenomena underlying the observation of Palaeolithic paintings.

As mentioned, art is a complex skill that requires high-demanded perceptual and attentional abilities (Bailey-Ross et al., 2019; Massaro et al., 2012). In this sense, both neuroscience and archaeology together can provide evidence about how the human visual system works in relation to the early manifestations of that skill (Onians, 2024). Recently, distinct studies have integrated behavioural sciences to understand the archaeological record (e.g., Criado-Boado et al., 2019; Meyering et al., 2020; Silva-Gago et al., 2023; Wisher et al., 2023b, c), but none have examined attentional distribution patterns across a diverse sample of depictions from the Cantabrian seacoast and other sites. The objective of this preliminary study was to analyse visual behaviour associated to Palaeolithic rock art, offering a methodology to quantify the distribution of attention within these paintings. Overall, our results show that visual attention was mainly directed towards the depicted figures, particularly the heads of the animals. No significant influence of the cave surface or other distracting elements was found. Furthermore, visual behaviour was consistent regardless of the representation’s completeness or chronological classification.

A general limitation of this kind of studies is the reliance on photographs rather than 3D models or direct examination of the original locations of the paintings. However, these alternatives would not allow the inclusion of a sufficiently diverse sample. In this sense, it is important to note that conclusions should be considered as general, not focused on particular painting or cave. Nonetheless, the results may be conditioned by the selection of caves and panels, although the sample was designed to be representative. Future analysis will incorporate more realistic environments, increasing the sample size, and exploring the level of visual scanning, including the assessment of informational complexity or entropy (Shiferaw et al., 2019).

## Supplementary Information

Below is the link to the electronic supplementary material.

Supplementary file1 (DOCX 1538 KB)

## Data Availability

Data is provided within the manuscript or supplementary information files.
