# Supplementary material for: Figure recognition and visual attention patterns behind the observation of Palaeolithic art
Source: J Cult Cogn Sci. 2025 Jun 8;9(2):225–41. doi: 10.1007/s41809-025-00170-0 (PMC12279578; doi:10.1007/s41809-025-00170-0)
Supplement: Supplementary file 1 — Supplementary file1 (DOCX 1538 KB) [file 41809_2025_170_MOESM1_ESM.docx]

**Supplementary material**

High resolution images can be downloaded from <https://saco.csic.es/s/FzcgJWRnXSQqKWs>


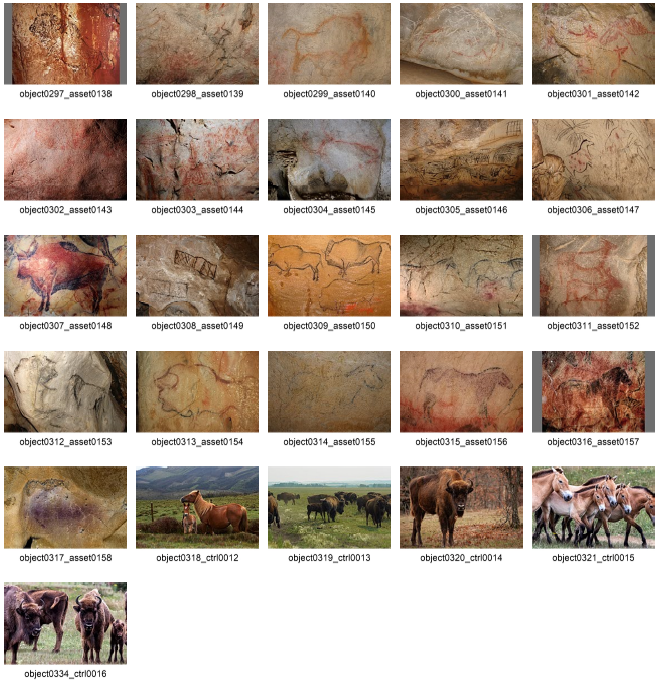


Figure 1. Experiment 1 stimuli


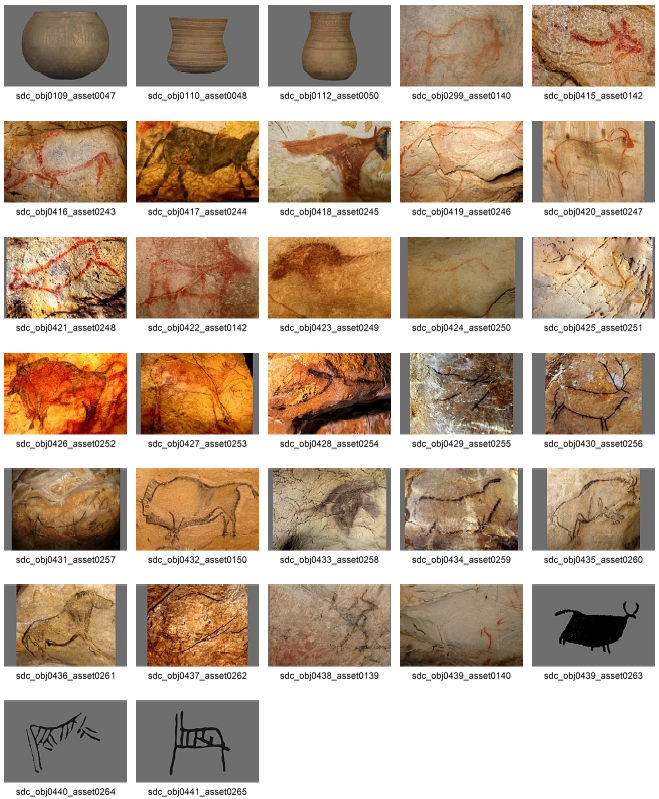


Figure 2. Experiment 2 stimuli


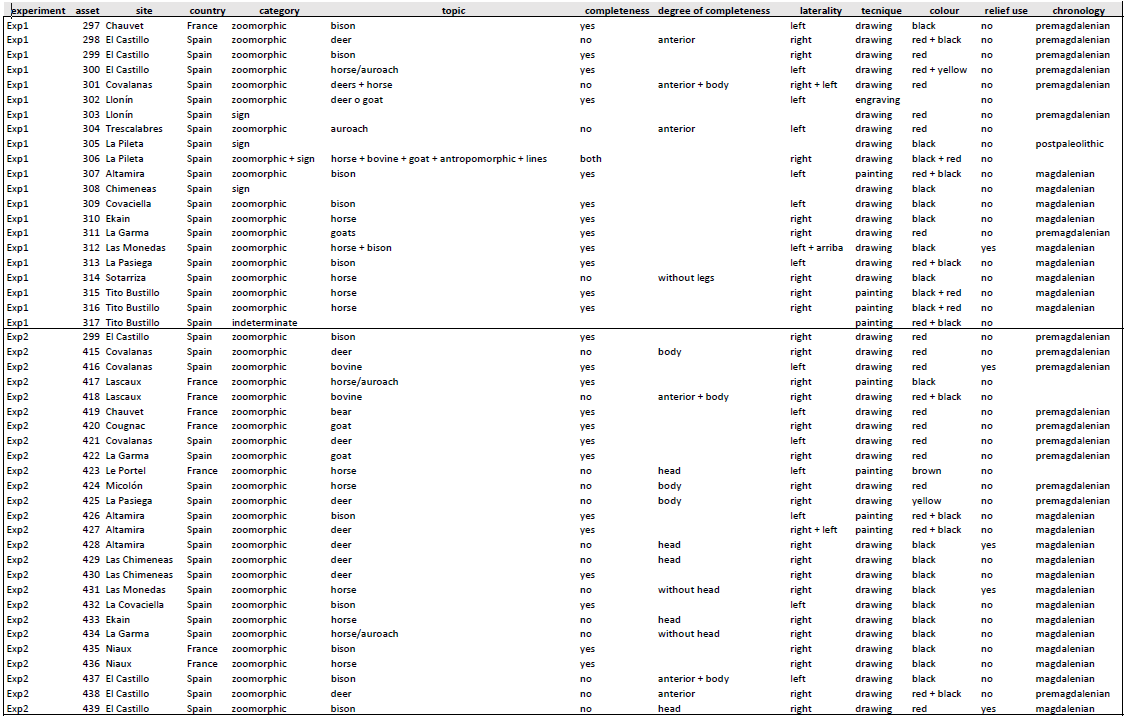


Table 1. Information about stimuli used in both experiments
